# Attitudes toward organizational change and their association with exhaustion in a sample of Italian police workers

**DOI:** 10.3389/fpsyg.2023.1122763

**Published:** 2023-03-20

**Authors:** Lara Colombo, Daniela Acquadro Maran, Annalisa Grandi

**Affiliations:** Department of Psychology, University of Turin, Turin, Italy

**Keywords:** police officers, burnout, change beliefs, resistance to change, role, gender

## Abstract

**Introduction:**

Change management is an important topic for organizations and some personal characteristics may help or hinder coping with change.

**Methods:**

The aim of the present study was to find out whether attitudes toward organizational change can predict work-related exhaustion in a sample of police workers (*N* = 532) in northern Italy. Differences between groups in relation to role (police officers vs. other roles) and gender were also considered.

**Results:**

The results of hierarchical regression showed that change beliefs were negatively associated to exhaustion, while resistance to change was positively related to exhaustion; role and gender were also significantly and positive associated with the dependent variable. Regarding differences between groups, police officers had lower positive change beliefs and higher levels of exhaustion compared to workers in other roles. Regarding gender, women reported higher levels of exhaustion than men.

**Discussion:**

The results of the present study provide further insights into what aspects should be considered when promoting organizational change in the police work context.

## Introduction

For an organization’s employees, change in one of its defining elements can be a source of despair. As the large body of research on organizational change demonstrates, workers may experience a period of uncertainty that affects their perceptions of what is happening in the workplace. As Dunford et al. (2012) have noted, change processes such as leadership transitions, mergers, downsizing, and restructuring have been shown to lead to work stress and, when prolonged, even burnout (Hu and Schaufeli, 2011). Burnout is a well known phenomenon that occurred in health care sector workers. In May 2019, burnout was included in the latest version of the International Classification of Diseases (ICD-11), which is promoted by the World Health Organization (WHO; see Hillert et al., 2020). Burnout was introduced in the previous version (ICD-10) but is expanded here. It is defined as a syndrome arising from the chronic presence of unsuccessfully managed work stress and characterized by three dimensions: (1) feelings of lack of energy or exhaustion; (2) mental disengagement from work or feelings of pessimism or cynicism related to work; and (3) decreased occupational effectiveness. Burnout refers specifically to the work context and should not be applied to describe experiences in other areas of life. However, due to the diagnostic ambiguity of burnout syndrome among mental health researchers (Chirico, 2017; Chirico et al., 2021), burnout is often explained and associated with existing diagnostic categories such as stress-related disorders or a specific type of depression. One of the motive of stress is related to resistance to change. Employees who are predisposed to be resistant to change show greater maladaptation to organizational demands during times of change, which can lead to increased stress responses such as exhaustion (Turgut et al., 2016). Exhaustion is the main component of burnout and is defined as the depletion of an individual’s energies as a result of an imbalance between demands and resources at work (Demerouti et al., 2010). Exhaustion is an important outcome in the study of stress in organizations for several reasons: it is thought to occur at an early stage of burnout development, thus providing an opportunity for intervention; it is both highly affective and chronic, suggesting that it is an appropriate indicator of cumulative work stress; and it has been shown to be more applicable to a variety of different jobs than other dimensions of burnout (Schaufeli and Peeters, 2000). In addition, exhaustion has been shown to be responsive to changes in an employee’s work environment, such as being new to the organization or changing jobs (Dunford et al., 2012).

Armenakis et al. (2007) identified five components that may influence the organizational belief that could cause resistance to change. The first is discrepancy, defined as the difference between the current state and an ideal or desired state. When organizational members do not know that the current state must change and that a different state is inevitable, fear of the unknown creates resistance. The second component is appropriateness: even if organizational members agree with the need for change, they may not agree with the proposed change initiative (Self and Schraeder, 2009). However, there must be agreement not only that the proposed change initiative is appropriate, but also that the initiative is consistent with the organization’s culture, structure, formal systems, etc. (Shimoni, 2017). The third component is social support. When a change is announced, employees turn to their immediate supervisor to learn the importance of the change. If the immediate/principal supervisor is also unaware of the reasons for the change, the readiness of both the member and the supervisor could be affected. Colleagues are also important in making sense of a proposed change initiative. Perceived support can be a boundary condition that can influence employee responses to change. While the impact of organizational change and the level of support experienced during the gradual implementation of a change process are likely to vary from unit to unit, members of the same work unit are generally exposed to common influences and experiences (Klein and Kozlowsk, 2000). Consequently, members of a work unit develop shared perceptions of the environment, creating a unique social context (Anderson and West, 1996; Tucker et al., 2013) that influences individual responses to change (Rafferty and Jimmieson, 2010). Therefore, it is plausible that general perceptions of support from a work unit are critical features of the change situation. Previous research has shown that perceived organizational support is influential at an aggregate level (Jin and Zhong, 2014). Therefore, it seems likely that it may also be important at the work unit level in the context of organizational change. If the work unit has a shared understanding that the organization cares about employees’ opinions and well-being and supports them during times of change, this may reduce anxiety about change and its consequences. Consequently, resistance to change as a character trait may be eliminated, and perceived nonconformity may reduce emotional exhaustion. The fourth message component proposed by Armenakis et al. (2007) is that of efficacy, the ability to produce the desired effects (Bandura and Locke, 2003, p. 87), which provides information needed to successfully implement a change initiative. This is important because members may lack confidence that the change can be successfully implemented. If management has failed to prepare the organization for change in the past, this may result in organizational members not only lacking confidence in themselves that they can successfully implement a change, but also lacking confidence in management’s ability to lead them in implementing the change. The final component of the change message to create readiness is valence. When confronted with a change in their current situation, organizational members resist the change if they see no benefit in the change or if the pain of the change outweighs the benefits of the change. On the other hand, if it can be demonstrated that the change will be more beneficial to the member, at least in the long run, he or she will be more likely to accept the change. This evaluative judgment about the change is a key component of valence. Even if management has demonstrated that a particular change initiative is necessary and that the organization will be better off as a result, and even if the organizational member acknowledges this, he or she will focus on how the change will affect him or her.

It is important to note that employee attitudes toward change can vary greatly from person to person, as they are the result of a complex interaction of emotions and cognitive processes. While some employees perceive organizational change as a means of rejuvenation, development, improvement, and growth, others may perceive it as instability, disorder, unpredictability, and risk (Cochran et al., 2002); as a result, organizational change may elicit undesirable reactions such as stress, cynicism, and resistance (Armenakis and Bedeian, 1999). Even when employees are aware of the need for change, they may resist it. This is because employees believe that they will lose something valuable as a result of the change. For example, change may cause resistance if it threatens an employee’s self-interest (van Dijk and van Dick, 2009). According to Oreg (2003), dispositional resistance to change reflects a person’s tendency to resist or avoid change, to generally dislike change, and to find change unpleasant in different contexts and types of change. Oreg (2003, 2006) describes resistance to change in terms of the following: (a) Behavioral (seeking routine, i.e., the extent to which a worker seeks routine and stable environments); (b) Affective (emotional response to imposed change, reflecting the extent to which workers find change uncomfortable and stressful, and short-term focus, reflecting the extent to which workers engage with short-term challenges as opposed to the long-term benefits of change); and (c) Cognitive rigidity, which is the reluctance to consider and test new perspectives and concepts.

### Police officers: Contextual characteristics and resistance to change

In the context of the police, change may be particularly difficult. Because of the nature of their work, police officers deal with many different situations involving compliance with the law, handling emergencies, cases of violence and personal injury, and so on. These situations make this a particularly vulnerable profession for chronic stress. Change can be an additional source of stress, a supplement to the complicated situations they deal with internally (e.g., bureaucracy) and externally (e.g., legislation) (Antoniou, 2009). As noted in previous research, work-related stress is felt more by women and individuals at a low hierarchical level. This condition is a burden because it makes the demands of the job seem particularly high. Although studies suggest that being a woman is not a risk factor *per se*, being a woman may be a risk factor for high levels of stress in certain occupations, such as police officers (Antoniou, 2009; Magnavita et al., 2018). In examining gender differences in occupational stress and burnout among police officers, McCarty et al. (2007) found that men and women did not report significantly different levels. However, their study suggests that there may be different predictive factors, with female officers being exposed to particular stressors. Burke et al. (2006) found little difference in work attitudes, satisfaction and mental health in a study of police in Norway, although female officers reported more psychosomatic symptoms. As for the lower ranks of the hierarchy, they are in charge of detecting traffic accidents, first intervention in cases of domestic violence, detecting violations, etc. Thus, it is they who are at the forefront of immediate intervention. Overall, Ahmadi and Zolikani (2022) examined the effects of cognitive flexibility on resistance to organizational change among 233 police officers in Mazandaran province. The results show that among these occupational groups, cognitive flexibility has a significant impact on resistance to organizational change.

In addition, it is interesting to note that resistance to change among police officers has been studied mainly when it comes to the introduction of technology, e.g., in the case of body-worn cameras (see Lum et al., 2020), when introducing evidence-based practices (see Koziarski and Kalyal, 2021), when reorganizing the way staff work and their tasks (see Gozzoli et al., 2018), and when improving gender equality in the career process (see van den Brink and Benschop, 2018). An interesting topic, however, is the change in leadership of a police organization. While most research has addressed the influence and role of a leader in resisting change (see Oreg and Berson, 2011; Rehman et al., 2021; Haesevoets et al., 2022), little attention has been paid to resistance to change when a leader changes (Northouse, 2021).

To address this gap in the literature, the purpose of this study was to analyze resistance to change in the municipal police organization during commander change. This police force was established in Italy more than 200 years ago. The municipal police is not included in Legislative Decree 121/181 and, according to the subsequent Law 65/1986, the local police forces contribute only in an auxiliary way to support the operations of the other forces of order (such as the State Police). The crimes typically prosecuted by the municipal police are those related to construction, ecology, environmental and landscape protection, hygiene and food, disturbance of public peace, and all crimes related to road traffic. It rarely investigates homicides and never criminal organizations, as they cannot operate outside its jurisdiction. The organization in which the research was conducted is located in a large city (more than 900 thousand inhabitants) in the northwest of Italy. As in all major Italian cities, in the municipal police, officers work in shifts, so that their services are available 24 h a day, 7 days a week. At the time of the study (2018), there were about 1800 employees in this police organization. It should be noted that during the study period, the retired commander was about to be replaced by a new commander from another police organization. It should be noted that other Italian studies have investigated work-related stress in this population in relation to stress and mental health (Garbarino et al., 2012, 2013, 2019; Setti and Argentero, 2013; Garbarino and Magnavita, 2015, 2019; Civilotti et al., 2022) and, more generally, absenteeism (Magnavita and Garbarino, 2013) and personality traits (Garbarino et al., 2012, 2014; Chiorri et al., 2015). To our knowledge, this is the first study conducted with the goal of analyzing police officers’ attitudes toward change at a time when a commander is changing.

## Current work

According to job demand–resources theory (JD-R) (Bakker and Demerouti, 2014), resources and demands are key aspects of any job. Demands entail affective, psychological, or physical effort, while resources are features of the job that promote goal attainment and personal growth, and thus can mitigate the negative effects of demands. Resources can be organizational, social, or personal in nature. In work contexts where change is occurring, workers’ personal attitudes toward change can be an element that, if present, can facilitate the process in a harmonious way. In contrast, the absence of a positive disposition, such as resistance to change, can create very critical working conditions.

Therefore, the objective of this study is twofold: to understand the differences in workers’ attitudes toward organizational change and in work-related exhaustion, and to determine which attitudes toward organizational change predict exhaustion. Our hypotheses are:

*H1*: Change beliefs, resistance to change and exhaustion differ in relation to sociodemographic characteristics (gender) and job role (police officer vs. other roles).

*H2*: Change beliefs (discrepancy, appropriateness, efficacy, principal support and valence) are negatively related to exhaustion.

*H3*: Resistance to change (routine seeking, emotional reaction, short-term focus and cognitive rigidity) is positively related to exhaustion.

## Materials and methods

The present study was carried out according to the guidelines of the Declaration of Helsinki (and subsequent revisions) and the ethical requirements of Italian legislation. No further ethical approval was obtained because no interventions or other medical procedures were foreseen that could cause biological, psychological, or social harm to the participants involved.

### Participants and procedures

To better understand the attitudes of the workers after organizational changes (determined by the change of the police commander), an *ad hoc* questionnaire with a short socio-demographic form was created and administered in presence among Northern Italy Municipal Police workers. The paper version of the questionnaire was accompanied by a form explaining the objectives of the study and the processing of the data (according to EU Regulation 2016/679). To participate in the study, workers had to read and sign the consent form. The questionnaire was anonymous, and participants did not receive any compensation or benefits. A total of 532 questionnaires were collected and used for the analyses. Most of the sample consisted of men (56.6%) and had a mean age of 51.41 years (range = 29–65, SD = 7.42) (see Table 1). Regarding marital status, 68.4% were married or cohabiting with a partner and 76.9% reported having children. Job tenure was 23.62 years (range = 5–43, SD = 8.68) and 56.8% of the sample were police officers. The rest of the sample were non-commissioned officers supporting the work of police officers, officers responsible for a service, unit managers responsible for implementing and monitoring outcomes within a unit and executives.

**Table 1:** Descriptive statistics of the sample (*N* = 532).

	*N*	%
Age (*M* = 51.41; SD = 7.42)		
Job tenure (*M* = 23.62; SD = 8.68)		
*Gender*		
Female	225	42.3
Male	301	56.6
Missing	6	1.1
*Relationship status*		
Single	82	15.4
Married/cohabiting	364	68.4
Separated/divorced	79	14.8
Widow/widower	3	0.6
Missing	4	0.8
*Children*		
Yes	409	76.9
No	121	22.7
Missing	2	0.4
*Role*		
Police officers	302	56.8
Other roles	225	42.3
Missing	5	0.9

### Measures

To investigate the hypotheses of the present study, validated measurement scales with good internal consistency were identified and included in the questionnaire.

#### Organizational change beliefs

In order to assess workers’ opinions about organizational change, the *Organizational Change Recipients’ Beliefs Scale*–*OCRBS* (Armenakis et al., 2007) was used. It consists of 24 items on a 5-point Likert scale (1 = never, 5 = always), and participants were asked to reflect on the period of the past 12 months. The OCRBS has 5 dimensions: discrepancy (4 items; a sample item is: “We needed to change the way we did some things in this organization”; Chronbach’s α = 0.86), appropriateness (5 items; a sample item is: “I believe the change will have a favorable effect on our operations”; Chronbach’s α = 0.86), efficacy (5 items; a sample item is: “I have the capability to implement the change”; Chronbach’s α = 0.77), principal support (6 items; a sample item is: “The top leaders support the change”; Chronbach’s α = 0.72) and valence (3 items; a sample item is: “With this change in my job, I will experience more self-fulfillment”; Chronbach’s α = 0.88). For the purposes of this study, also the general index was used, and Chronbach’s α was 0.90. One item (No. 24) was excluded from the index because it did not fit the work context studied.

#### Resistance to organizational change

To assess workers’ disposition to resist changes, the *Resistance to Change Scale* (Oreg, 2003) was used. The scale includes 17 items on a 5-point Likert scale (1 = strongly disagree, 5 = strongly agree). The *Resistance to Change Scale* has 4 dimensions: routine seeking (5 items; an example item is: “I’d rather be bored than surprised”; Chronbach’s α = 0.70), emotional reaction (4 items; an example item is: “When I am informed of a change of plans, I tense up a bit”; Chronbach’s α = 0.80), short-term focus (4 items; an example item is: “Changing plans seems like a real hassle to me”; Chronbach’s α = 0.79), and cognitive rigidity (4 items; an example item is: “I do not change my mind easily”; Chronbach’s α = 0.70). For the purposes of this study, the general index of the scale was also used. The Chronbach’s α of the overall scale in this study was 0.83.

#### Exhaustion

To assess workers’ physical, cognitive, and affective levels of work-related exhaustion, the *Exhaustion* subscale of the Oldenburg Burnout Inventory–OLBI (Demerouti et al., 2010) was used. The scale includes 8 items on a 4-point Likert scale (1 = strongly disagree, 4 = strongly agree); an example item is: “There are days when I feel tired before I arrive at work.” The Chronbach’s α of the entire scale in this study was 0.74. Since there are no standard cutoff values for OLBI, the values reported by Block et al. (2020), i.e., M_EXH_ = ≥ 2.50, are considered as reference values.

### Data analysis

Descriptive statistical analyses (means and standard deviations of the scales, see Table 2) and reliability of the scales (Cronbach’s alpha) were performed using IBM SPSS 27 (Statistical Package for Social Science). Pearson correlations between all variables were calculated. Hierarchical linear regression analyses were also conducted to determine the role of organizational change and resistance to change as predictors of exhaustion. In the regression model, multicollinearity between variables was assessed using the variance inflation factor (VIF): no multicollinearity problem was found (VIF < 5). The differences between groups in the means of the variables were analyzed using analysis of variance (*t*-tests for independent samples).

**Table 2:** Means, standard deviations of the scales and observed ranges for each variable.

	Mean	SD	Range
**Outcome**			
Exhaustion	2.35	0.35	1–4
**Organizational change beliefs**			
(*General index*)	3.22	0.47	1–5
Discrepancy	3.66	0.14	1–5
Appropriateness	3.01	0.38	1–5
Efficacy	3.61	0.39	1–5
Principal support	2.87	0.45	1–5
Valence	3.03	0.17	1–5
**Resistance to change**			
(*General index*)	2.45	0.58	1–5
Routine seeking	2.10	0.27	1–5
Emotional reaction	2.35	0.14	1–5
Short-term focus	2.10	0.15	1–5
Cognitive rigidity	3.36	0.38	1–5

## Results

### Differences between groups

#### Organizational change beliefs

*Change beliefs* total score was slightly above the average scale score (*M* = 3.22, SD = 0.47), with lower positive belief among police officers (*M* = 71.94, SD = 13.69) than for all other roles (*M* = 76.74, SD = 12.99), *t* (4.068) = 525, *p* < 0.001, Cohen’s D = 0.36. No significant differences were found with respect to gender.

With regard to the subscales, perceptions of *discrepancy* were above the average scale score (*M* = 3.66, SD = 0.14), with lower values among police officers (*M* = 14.39, SD = 3.62) than for all other roles (*M* = 14.97, SD = 3.41), *t*(1.847) = 525, *p* = 0.03, Cohen’s D = 0.16. No significant differences were found with respect to gender.

*Appropriateness* score was in line with the average scale score (*M* = 3.01, SD = 0.38), with lower values among police officers (*M* = 14.46, SD = 4.62) than for all other roles (*M* = 15.82, SD = 3.96), *t*(3.627) = 514.66, *p* < 0.001, Cohen’s D = 0.31. No significant differences were found with respect to gender.

Perceptions of *efficacy* were above the average scale score (*M* = 3.61, SD = 0.39), with lower values among police officers (*M* = 17.73, SD = 3.58) than for all other roles (*M* = 18.40, SD = 3.51), *t*(2.144) = 525, *p* = 0.02, Cohen’s D = 0.19. No significant differences were found with respect to gender.

*Principal support* score was slightly below the average scale score (*M* = 2.87, SD = 0.45), with lower values among police officers (*M* = 16.71, SD = 3.98) than for all other roles (*M* = 17.89, SD = 4.02), *t*(3.349) = 525, *p* < 0.001, Cohen’s D = 0.29. No significant differences were found with respect to gender.

*Valence* was in line with the average scale score (*M* = 3.03, SD = 0.17), with lower values among police officers (*M* = 8.64, SD = 2.81) than for all other roles (*M* = 9.65, SD = 2.93), *t*(4.028) = 525, *p* < 0.001, Cohen’s D = 0.35. No significant differences were found with respect to gender.

#### Resistance to change

*Resistance to change* total score was below the average scale score (*M* = 2.45, SD = 0.58), with no significant differences found with respect to role and gender.

With regard to the subscales, *routine seeking* score was below the average scale score (*M* = 2.10, SD = 0.27), with no significant differences found with respect to role and gender.

*Emotional reaction* was below the average scale score (*M* = 2.35, SD = 0.14), with no significant differences found with respect to role and gender.

*Short-term focus* was below the average scale score (*M* = 2.10, SD = 0.15), with no significant differences found with respect to role and gender.

*Cognitive rigidity* was above the average scale score (*M* = 3.36, SD = 0.38), with no significant differences found with respect to role and gender.

#### Exhaustion

Self-reported feelings of *exhaustion* were above the average scale score (*M* = 2.35, SD = 0.35) and slightly below the cutoff point, with higher scores for police officers (*M* = 19.22, SD = 4.32) than for all other roles (*M* = 18.12, SD = 4.04), *t*(−2.962) = 525, *p* = 0.002, Cohen’s D = 0.26. In relation to gender, women showed higher levels of exhaustion (*M* = 19.17, SD = 4.25) than men (*M* = 18.51, SD = 3.91), *t*(−1.743) = 441.41, *p* = 0.04, Cohen’s D = 0.16.

### Correlations and regressions

In the sample, correlations were calculated between exhaustion, change beliefs and resistance to change dimensions. All significant correlations were in the expected direction. Pearson coefficients are shown in Table 3. As to organizational change beliefs, all the measures but discrepancy had strong negative correlations with exhaustion (*p* < 0.01). As to resistance to change, all the measures but cognitive rigidity had strong positive correlations with the dependent variable (*p* < 0.01). Role (1 = police officers) was also found positively correlated with exhaustion (*p* < 0.01).

**Table 3:** Sample correlations (*N* = 532).

Measures	Mean (SD)	1	2	3	4	5	6	7	8	9	10	11	12	13
1. Exhaustion	18.77 (4.23)	–												
2. Gender (1 = women)	–	0.078	–											
3. Role (1 = p. officers)	–	0.128^**^	0.127^**^	–										
4. Job tenure	23.62 (8.68)	−0.078	−0.127^**^	−0.754^**^	–									
5. Routine seeking	10.48 (3.86)	0.183^**^	−0.060	0.063	−0.032	–								
6. Emotional reaction	9.40 (3.72)	0.320^**^	−0.022	−0.008	0.028	0.531^**^	–							
7. Short-term focus	8.39 (3.50)	0.241^**^	−0.047	0.019	0.008	0.536^**^	0.657^**^	–						
8. Cognitive rigidity	13.44 (3.27)	−0.054	−0.025	0.020	0.027	0.033	0.052	0.019	–					
9. Discrepancy	14.65 (3.54)	−0.025	−0.021	−0.080	−0.022	−238^**^	−0.070	−0.121^**^	−0.078	–				
10. Appropriateness	15.07 (4.40)	−0.275^**^	0.019	−0.153^**^	0.114^**^	−0.202^**^	−0.174^**^	−0.113^**^	−0.064	0.367^**^	–			
11. Efficacy	18.04 (3.57)	−0.319^**^	0.059	−0.093^*^	0.059	−0.290^**^	−0.269^**^	−0.291^**^	0.008	0.199^**^	0.546^**^	–		
12. Principal support	17.21 (4.02)	−0.308^**^	0.031	−0.145^**^	0.155^**^	0.025	−0.088^*^	−0.083	0.017	0.050	0.528^**^	0.534^**^	–	
13. Valence	9.08 (2.90)	−0.327^**^	0.041	−0.173 ^**^	0.149^**^	−0.216^**^	−0.186^**^	−0.115^**^	−0.096^*^	0.220^**^	0.670^**^	0.539^**^	0.559^**^	–

SD, standard deviation. ^**^p < 0.01; ^*^p < 0.05.

Hierarchical linear regression analyses (see Table 4) were conducted to understand whether change-related attitudes could predict exhaustion at work. Exhaustion was considered the dependent variable, and gender, role and length of service were included as control variables.

**Table 4:** Hierarchical multiple regression (exhaustion = dependent variable).

	*β*	*t*	*p*
Exhaustion			
**1st step (control variables)**			
Gender (1 = women)	0.070	1.589	0.113
Role (1 = police officers)	**0.174**	**2.625**	**0.009**
Job tenure	0.070	1.061	0.289
*R*^2^ = 0.02			
**2nd step (change beliefs)**			
Gender (1 = women)	**0.098**	**2.385**	**0.017**
Role (1 = police officers)	**0.124**	**2.001**	**0.046**
Job tenure	0.090	1.448	0.148
Appropriateness	−0.027	−0.460	0.646
Efficacy	**−0.157**	**−2.978**	**0.003**
Principal support	**−0.135**	**−2.546**	**0.011**
Valence	**−0.142**	**−2.395**	**0.017**
*R*^2^ = 0.16			
**3rd step (resistance to change)**			
Gender (1 = women)	**0.097**	**2.442**	**0.015**
Role (1 = police officers)	**0.124**	**2.062**	**0.040**
Job tenure	0.078	1.302	0.193
Appropriateness	−0.020	−0.359	0.720
Efficacy	−0.088	−1.651	0.099
Principal support	**−0.153**	**−2.910**	**0.004**
Valence	**−0.136**	**−2.348**	**0.019**
Routine seeking	−0.055	−1.102	0.271
Emotional reaction	**0.226**	**4.116**	**< 0.001**
Short-term focus	0.068	1.211	0.226
*R*^2^ = 0.22			

Boldface was used to highlight significant values.

Gender, role and job tenure were introduced as control variables in Step 1, and only role (*β* = 0.17, *p* = 0.009) showed a significant effect on exhaustion (2% explained variance). In Step 2, organizational change beliefs (appropriateness, efficacy, principal support and valence) were introduced. Among these variables, efficacy (*β* = 0.16, *p* = 0.003), principal support (*β* = 0.13, *p* = 0.011), and valence (*β* = 0.14, *p* = 0.017), were significantly and negatively associated with exhaustion; between the former introduced variables, role (*β* = 0.12, *p* = 0.046) and gender (*β* = 0.10, p = 0.017) were significantly and positively associated with the dependent variable. The variables added to the model were good predictors of the dependent variable since there was a significant change in *R*^2^ coefficient (16% explained variance). Finally, in Step 3, resistance to change dimensions (routine seeking, emotional reaction and short-term focus) were introduced. Among these variables, only emotional reaction (*β* = 0.23, *p* < 0.001) was significantly and positively associated with exhaustion; as to the organizational change beliefs dimensions, principal support (*β* = −0.15, *p* = 0.004), and valence (*β* = −0.14, *p* = 0.019), remained significantly and negatively associated with the dependent variable. Role (*β* = 0.12, *p* = 0.040), and gender (*β* = 0.10, *p* = 0.015), also remained significantly and positively associated with the dependent variable. The further change in *R*^2^ coefficient (22% explained variance) showed that the new variables were also good predictors of exhaustion. The *F* value showed a significant *R*^2^ change associated with each of the 3 steps.

## Discussion

The purpose of this paper is to describe the role of change beliefs and resistance to change on exhaustion in a sample of Italian police organization. The opportunity for this study arose from the rotation of the commander of this organization. Therefore, to better understand how police employees accept organizational change, beliefs about change and resistance to change were examined. Overall, the results of this study showed that police officers (those employees at the lowest rank in the hierarchy) tend to have lower attitudes toward change than employees in other hierarchical positions: from an organizational perspective, police officers describe that change will not bring anything new, and they describe that the organization is not able to support changes that will not affect their work. This finding suggests that they are afraid of what change might bring. As Nilsen et al. (2020) note, it is important for each employee to be involved early in the change process and to be able to influence throughout the process to ensure the success of the change. Otherwise, there is a risk that organizational change will fail. In the context of police organization, the risk is that police officers who feel they are not included in the change process will be dissatisfied at work, resulting in lower job performance (Paoline and Gau, 2020). The lower performance affects the ability to provide an effective response to citizens, potentially harming not the individual and the organization, but the citizens to whom the service is dedicated (Gutshall et al., 2017). It is interesting to note that resistance to change was reported for the organizational variables: The results on dispositional resistance to change showed that there was no difference by hierarchical role and gender. However, there was a difference in perceptions of exhaustion, where police officers were more inclined to report a high score than police officers in other roles, and women were more inclined to report a high score than men. Thus, Hypothesis 1 (change beliefs, resistance to change, and exhaustion differ as a function of sociodemographic characteristics – gender – and job role – police officers vs. other roles) was partially confirmed. These results confirmed that resistance to change and exhaustion differ among individuals in low hierarchical positions and among women, suggesting that those who perceive low autonomy are more reluctant to change (Battistelli et al., 2013).

Regarding hypothesis 2 (change beliefs – discrepancy, appropriateness, efficacy, principled support, and valence – are negatively related to exhaustion), the results showed that change beliefs, but not discrepancy, were related to exhaustion (hypothesis 2 was thus only partially confirmed). This result is particularly interesting. As described by Rafferty and Minbashian (2019), discrepancy is a belief that change is based on legitimate reasons and is necessary to address a deficiency in the current state compared to a desired future state. In the case of this police organization, the change in commander was indeed a necessity because the previous commander retired. It is possible that the situation of limbo (the old commander is no longer on duty and is waiting for the new commander) led to a situation of distrust in the organization in terms of its ability to cope with an anticipated change. This situation could increase feelings of uncertainty (Kern and Zapf, 2021), which could lead to negative feelings such as worries about the future, with possible consequences for perceptions of stress (Bottesi et al., 2019).

Hypothesis 3 referred to the relationship between resistance to change and exhaustion. The results show that cognitive rigidity is positively associated with exhaustion in police officers. As described below, cognitive rigidity refers to the tendency to avoid alternative ideas and different views of reality is the fear of the new way of thinking (Oreg, 2003; Mareš, 2018). Cognitive rigidity also includes the tendency to develop and persist in a particular cognitive pattern, even in situations where the pattern is no longer effective (Morris and Mansell, 2018). Moreover, in police organization, there is a tendency to assign certain roles to officers, and he/she may develop rigid thinking, which results in there being “only one” right way to accomplish a certain task (Johnson and Krawczyn, 2022). Therefore, it may be difficult to develop alternative solutions or perspectives when faced with emotional issues that involve change. The results of the hierarchical linear regression show that, of the beliefs about organizational change, efficacy, principled support, and valence are negatively associated with exhaustion. This suggests that workers who perceive support from their peers, coordinator, and management receive answers to their concerns and fears, share the effort that change entails, and receive useful feedback to address the proposed scenario. In addition, the perceived effectiveness in managing the change could be related to a previous similar situation that included the change of commander. A high position could be related to years of work experience in the police organization (Jamil et al., 2020). Thus, perceptions of effectiveness could be derived from previous experiences of success, experiences that police officers may lack. Experience within the police organization and high rank within that organization (usually achieved by men) could explain the valence and lower expression of exhaustion. Communication between higher levels of the hierarchy may facilitate the exchange of necessary information about the possible scenarios that a change may entail and foresee benefits such as making demands in favor of one’s position and/or work unit. As suggested by Yun et al. (2020), positive communication is associated with less exhaustion, while negative communication is associated with more exhaustion. On the resistance to change dimension, emotional response was significantly and positively associated with exhaustion. Emotional reaction is the affective component of resistance to change: These emotions can cause anger, frustration, and trigger stress that can lead to expressing negative feelings or opinions about the change to others (Nilsen et al., 2019). This expression could be an adaptive coping strategy for the employee and at the same time an maladaptive one for the group, as the adverse effects of the negative emotions are exacerbated by the expression (see Brown et al., 2005).

This study, of course, has some limitations. First, it was a cross-sectional study and the sample belongs to a single organization, so the results should be taken with caution and cannot be generalized. Future research could include a larger sample and comparison across organizations. This could also help to understand how change is experienced in the context of the leadership style that has shaped the organization up to this point. Leadership appears to be key to understanding the success or failure of change in organizations: in their study, Vos and Rupert (2018) found that leadership behavior can influence the increase or decrease in resistance to change. A longitudinal study could help to better understand the moment of change from one commander to another, what the expectations, doubts, and concerns are, and identify different leadership styles and their effects on police workers emotions and behaviors (Carleton et al., 2020). In addition, a longitudinal study could help understand perceived stress during the pandemic: The effort required of police officers was higher than during “normal” times, and leadership styles could affect how the emergency period was managed (Stogner et al., 2020). It should be noted that the results refer to a specific period in which the commander changes in this sample. Further research could look at organizational changes over the past 10–20 years and how societal changes have affected organizational changes. For example, migration flows have reshaped cities, as have new housing needs and the types of services provided. In addition, our study focused on participants’ general attitudes toward organizational change when contextualizing their responses. A more comprehensive survey could include a focus on their current work experiences: for example, interviews and focus groups could be used to explore in depth the meaning of organizational change, considering opportunities and potential drawbacks. It might also be useful to examine in more detail how attitudes toward change are related to the level of education (e.g., provided by the organization, such as a specific training course) or the various roles and services offered to citizens. Further research could also consider these variables to better explain attitudes toward change in this population. Additionally, due to the quantitative nature of the study, it was not possible to collect data on the organizational and subculture that characterized this police organization. As Cohen (2017) argues, there is a strong relationship between employee values, organizational culture, and the success or failure of organizational change efforts (see also Terpstra and Salet, 2019). The way police workers are selected, trained, and guided and the organizational climate influence perceptions of events and even organizational change (Alpert et al., 2012). Future research could use a qualitative or mixed methods approach to further examine organizational culture and climate and their influence on resistance to change (both in terms of beliefs about organizational change and dispositional resistance to change). Another limitation is that we did not ask about some sociodemographic variables related to race or ethnicity, variables that could help explain the relationship within the organizational context and thus attitudes toward change. Finally, there may be a bias related to socially desirable response behavior, i.e., the tendency to respond to a questionnaire while projecting a positive image of oneself. As Xavier et al. (2021) noted, police officers may tend to reduce complex issues and align their narratives with what is considered socially acceptable or desirable for the image they portray to the public. Future research could incorporate a social desirability scale to examine how the image of the police organization and the desire to conform to social norms may have influenced police officers’ responses.

Despite these limitations, the results of this study may contribute to a better understanding of organizational and dispositional resistance to change. Specifically, the findings suggest that management must monitor the process, provide information, ensure transparency in the decision-making process, and involve police employees in every step of the change process. Following Warrick (2022), an open dialog could be helpful in managing the process by first developing a safe culture where there is an opportunity to create a space where all stakeholders can contribute equally and express fears and concerns about the change. Open dialog could first be encouraged by managers who lead by example and participate in openness. Then, it is necessary to create opportunities for open dialog, for example, by organizing meetings where employees can reflect on the change, conducting surveys, and providing feedback. In addition, sharing information can keep employees appropriately informed about change, which improves open dialog about impact in the organization. In police organizations, open dialog could be particularly useful in improving an organizational culture that focuses on expressing feelings and overcoming the stigma associated with asking for help and emotional support, as well as the social desirability associated with wearing the uniform.

## Data availability statement

The raw data supporting the conclusions of this article will be made available by the authors, without undue reservation.

## Ethics statement

Ethical review and approval was not required for the study on human participants in accordance with the local legislation and institutional requirements. The patients/participants provided their written informed consent to participate in this study.

## Author contributions

DA, AG, and LC: conceptualization, investigation, writing—review and editing, and writing—original draft preparation. LC and AG: methodology and data analysis. All authors contributed to the article and approved the submitted version.

## Conflict of interest

The authors declare that the research was conducted in the absence of any commercial or financial relationships that could be construed as a potential conflict of interest.

## Publisher’s note

All claims expressed in this article are solely those of the authors and do not necessarily represent those of their affiliated organizations, or those of the publisher, the editors and the reviewers. Any product that may be evaluated in this article, or claim that may be made by its manufacturer, is not guaranteed or endorsed by the publisher.
